# Molecular immune mechanisms of HPV‐infected HaCaT cells in vitro based on toll‐like receptors signaling pathway

**DOI:** 10.1002/jcla.23101

**Published:** 2019-11-29

**Authors:** Zuolin Ying, Xiaojie Li, Hong Dang, Na Yin, Chuang Gao

**Affiliations:** ^1^ Department of Dermatology Shanghai General Hospital Shanghai Jiao Tong University School of Medicine Shanghai China

**Keywords:** HPV‐infected HaCaT cells, molecular immune mechanisms, toll‐like receptors

## Abstract

**Objective:**

To explore the molecular immune mechanism of HPV‐infected HaCaT cells in vitro based on TLRs signaling pathway by analyzing the effects of interfering TLRs on inflammatory and immune factors in the signaling pathway.

**Methods:**

FCM was used to analyze the proportion of Th1, Th2, Th17, and Treg cells in blood samples. HPV‐infected HaCaT cells were divided into five groups: A, B, C, D, and E. Group A added TLR3 antagonist, group B added TLR9 antagonist, group C added equivalent saline, group D added IRF3 agonist, and group E added IRF3 inhibitor. Immunohistochemistry was used to analyze the expression of TLR3 and TLR9 in HaCaT cell model; ELISA was used to analyze the expression of inflammatory factors IL‐2, TNF‐a, and IFN‐beta; WB was used to analyze the expression of TRAF3, IKK epsilon, and TBK1; RT‐PCR was used to analyze the expression of IRF3 and IRF7 in each cell model.

**Results:**

The proportion of blood immune cells in patients with HPV infection was Th1, Th17, Th2, and Treg, with statistical significance (*P* < .05); the expression of TLR3 and TLR9 in HPV‐infected cells was higher than that in negative control group, with statistical significance (*P* < .05); TLR3 was higher than TLR9, with no significant difference (*P* > .05); the expression of IL‐2, TNF‐alpha, IFN‐beta in each group, TLR3, and TLR9 was higher than that in negative control group (*P* < .05). The expression of TRAF3, IKK epsilon, and TBK1 in the control group was higher than that in the TLR3 and TLR9 inhibitor groups, and the expression of IRF3 and IRF7 in the TLR9 inhibitor group was higher than that in the TLR3 inhibitor group (*P* < .05); the expression of IRF3 and IRF7 in the TLR3i and TLR9i inhibitor groups was lower than that in the TLR3 inhibitor group (*P* < .05). Compared with the control group, IRF3a group was higher than the control group, IRF3i group was lower than the control group, the difference was statistically significant (*P* < .05).

**Conclusion:**

TLR3 and TLR9, the key factors of TLRs, are highly expressed in HaCaT cells infected with HPV. Through TLRs‐IKK‐e‐IRFs‐IFN signaling pathway, they can induce high expression of inflammatory factors, IKK‐e, IRFs, and IFN, and improve immunity.

## 1 INTRODUCTION

Human papillomavims (HPV) is a class of non‐enveloped DNA viruses with strict host range and tissue specificity. HPV is closely related to many human diseases. Condyloma acuminatum (CA) is a benign papillomatous proliferative disease caused by human papillomavirus (HPV) infection in human genital parts and adjacent epidermis. The treatment is not difficult, but the recurrence rate is high, and the incidence is increasing year by year.^1^, ^2^ It has been suggested that the expression of TLR3 and TLR9, the typical factors of Toll‐like receptors (TLRs), is related to the clearance of HPV16 virus, but the mechanism is still unclear.^3^ TLRs are a family of transmembrane signal transduction receptors that mediate innate immunity. They can recognize the conservative molecular components of specific microorganisms, that is pathogen‐related molecular patterns, and resist the invasion of foreign pathogenic microorganisms.^4^ TLR3 and TLR9 are considered as one of the important receptors of antiviral immunity. TLR signaling pathway involves many factors such as interferon regulatory factors (IRFs), IKK epsilon/TBK1, interferon regulatory factors (IRFs), and interferon (IFN).^5^ Studies have shown that the activation of TLRs receptor can activate the immune signaling pathway of TLRs‐IKK epsilon‐IRFs‐IFN in host cells and induce immune inflammation. The immune inflammatory factors produced can activate immune cells Th1, Th2, Th17, and Treg cells and produce immune cascade effect.^6^, ^7^ In this study, the expression of TLRs signaling pathway‐related factors, immune factors, and inflammatory factors in HaCaT cells infected with HPV was determined to explore the molecular immune mechanism of HPV‐infected HaCaT cells in vitro based on TLRs signaling pathway.

## 2 MATERIALS AND METHODS

### 2.1 Main instruments and reagents

HaCaT cell line, Research Area Biotechnology (Shanghai) Co., Ltd., fetal bovine serum FBS and RPMI1640 culture medium (Nanjing Dean Biotechnology Co., Ltd.). LipofectamineRNAiMAX (American Invitrogen Company), reverse transcription, Real‐time PCR (American ABI Company); IL‐2, TNF‐alpha, IFN‐beta ELISA kit (Nanjing Dean Biotechnology Co., Ltd.); BCA protein level kit (American Thermo Company), beta‐actin antibody (American CST Company), mouse anti‐human TRAF3, IKE, and TBK1 antibody‐anti (American Jacks). HRP labeled goat anti‐rabbit and anti‐mouse antibody (Jackson Company) and hypersensitive ECL (Pierce Company).

Digital camera (model Penguin 600CL Pixera Inc), Leica full automatic microscope (model DMLA, Leica Inc), and software Simple PCI (V5.2, Compix Inc).

### 2.2 Method

#### 2.2.1 Cell culture

HaCaT cells were removed from the cold storage and resuscitated in a constant temperature water bath at 37°C. The suspension was added 2 mL 1640 medium containing 10% FBS, 1000 rpm/min, and centrifuged for 8 minutes. After centrifugation, the supernatant was removed and transferred to a incubator with 5% CO_2_ at 37°C.

#### 2.2.2 Experimental steps

1. Twelve pairs of blood samples were collected from patients with HPV infection and healthy persons. The proportion of immune cells Th1, Th2, Th17, and Treg in blood samples was analyzed by FCM.
2. HPV‐infected HaCaT cells and made cell models. The expressions of TLR3 and TLR9 in HaCaT cell models were analyzed by immunohistochemistry.
3. HaCaT cells were infected with HPV and divided into five groups: A, B, C, D, and E. Group A was treated with polyinosinic acid‐polycytidylic acid (PIC), group B was treated with CpG oligonucleotide, group C was treated with saline, group D was treated with IRF3 agonist, and group E was treated with IRF3 inhibitor.
4. The supernatants of each group were collected, and the expression levels of inflammatory factors IL‐2, TNF‐alpha, and IFN‐beta were analyzed by ELISA.
5. Proteins in each cell model of A, B, and C were collected, and the expression levels of TRAF3, IKK epsilon, and TBK1 were analyzed by WB.
6. RT‐PCR was used to analyze the expression of IRF3 and IRF7 in each cell model.

#### 2.2.3 Detection method

1. Immunohistochemical staining was used to take the cell model, fixed with 4% formaldehyde solution, embedded in paraffin, and sectioned continuously for 4 mol/L. After dewaxing to water, 3% hydrogen peroxide solution blocked endogenous peroxidase, antigen was repaired at 120 for 3 minutes, normal goat serum was closed for 10 minutes, primary antibody was added at 37°C for 1 hour, and biotin‐labeled secondary antibody and streptavidin‐peroxidase (SP) complex were incubated at 37°C for 30 minutes. DAB colouring, hematoxylin dyeing, dehydration, transparency, sealing. The known positive tablets were used as positive control, and PBS was used as negative control instead of primary antibody.
2. The results showed that immunohistochemical staining was positive for cytoplasm or membrane brown‐yellow staining, and strong staining according to positive cells.

Semi‐quantitative grading of TLR3 and TLR9 expression was determined by degrees and percentages: (a) According to the color intensity of cells, the colorless score was 0, the light yellow score was 1, the Yellow score was 2, and the brown‐yellow score was 3; (b) According to the percentage of positive cells, the percentage of positive cells was 0, 11%‐25% was 1, 26%‐50% was 2, 51%‐75% was 3, and >76% was 4. According to the product of the above two scores, 0 is negative (−), 1‐4 is weak positive (+), 6‐8 is positive (+), and 9‐12 is strong positive (++). Single‐blind method was used. Five high‐fold (400×) visual fields were randomly selected for each slice. The average score was used to judge the semi‐quantitative grade of the specimen. The positive rate of expression was equal to the number of positive specimens/the total number of specimens × 100%.

### 2.3 Statistical method

SPSS19.0 software was used to analyze the data. The measurement data were expressed in the form of mean ($\bar{x}$ ± s), the comparison between groups was expressed in the form of *t* test, the counting data were expressed in the form of percentage (%), and the comparison between groups was expressed in the form of chi‐square test. The statistical analysis showed significant difference in *P* < .05.

## 3 RESULTS

### 3.1 FCM analysis of the proportions of immune cells Th1, Th2, Th17, and Treg in blood samples

**Figure 1:**
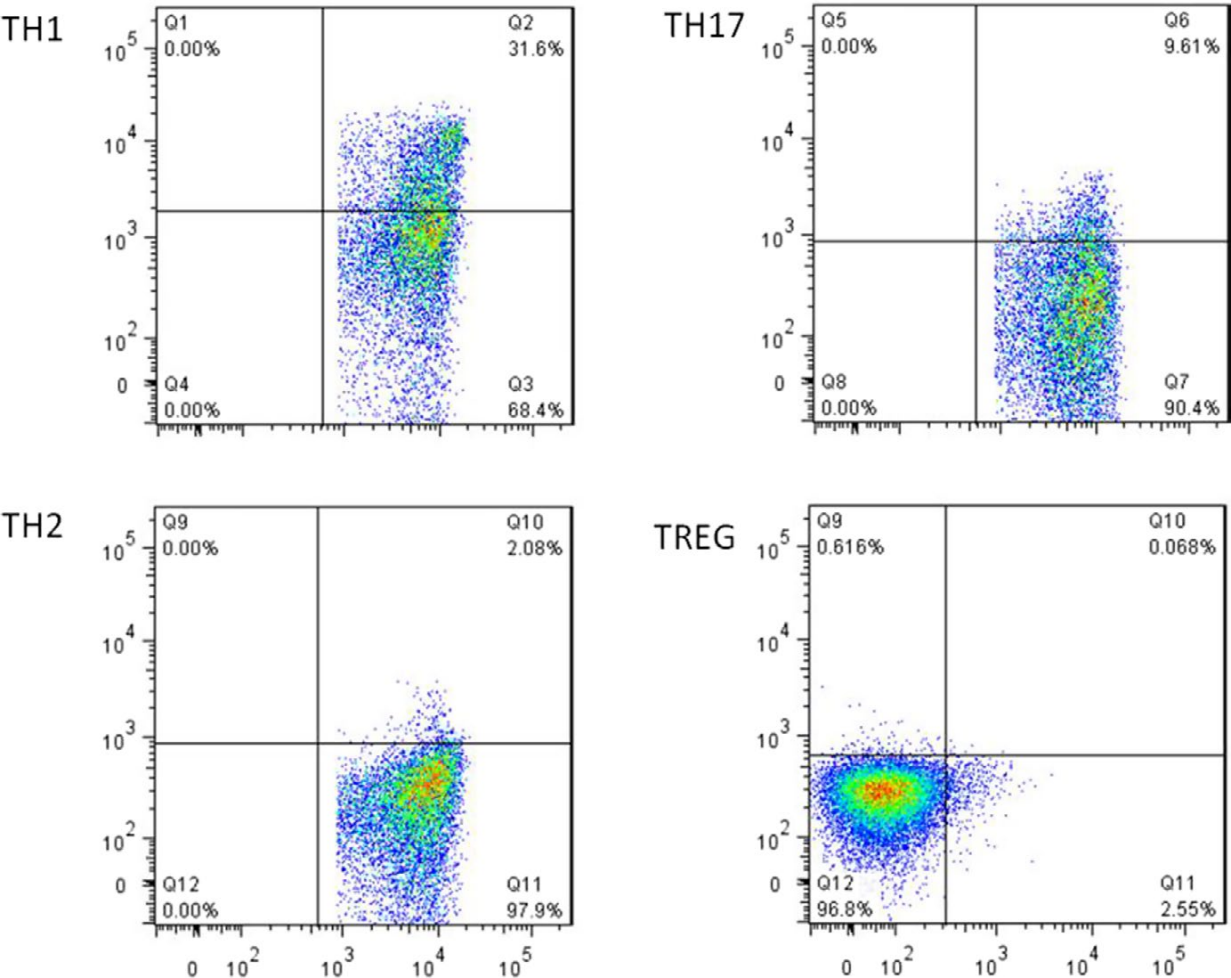
The proportion of blood immune cells in patients with HPV infection (Th1 > Th17 > Th2 > Treg)

The proportion of blood immune cells in patients infected with HPV was Th1, Th17, Th2, and Treg from high to low, with statistical significance (*P* < .05), as shown in Figure 1.

### 3.2 Immunohistochemical analysis of TLR3 and TLR9 expression in HaCaT cell model

**Figure 2:**
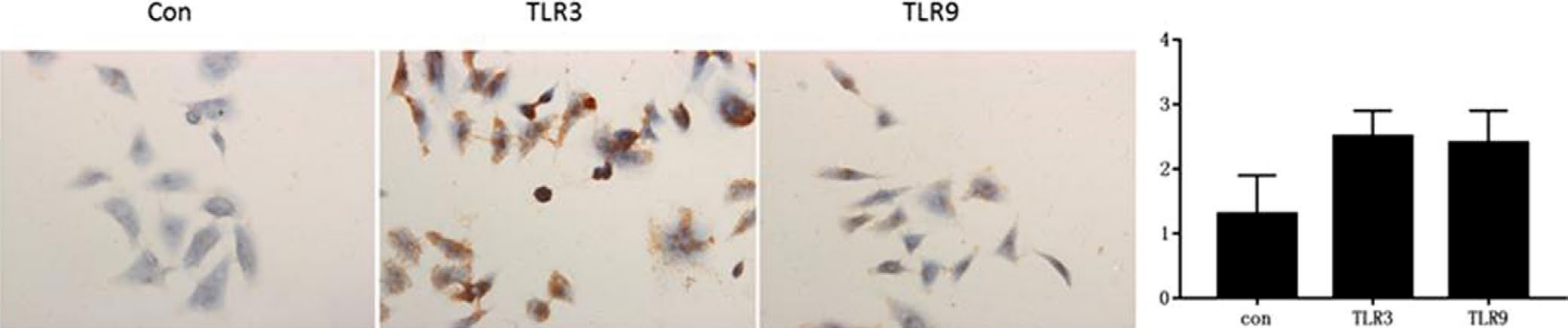
The expression of TLR3 and TLR9 in HaCaT cell model

The expression levels of TLR3 and TLR9 in HPV‐infected cells were higher than those in the negative control group, with statistical significance (*P* < .05), while TLR3 was higher than TLR9, with no significant difference (*P* > .05), as shown in Figure 2.

### 3.3 ELISA analysis of the expression of inflammatory factors IL‐2, TNF‐alpha, and IFN‐beta in HaCaT cell model

**Figure 3:**
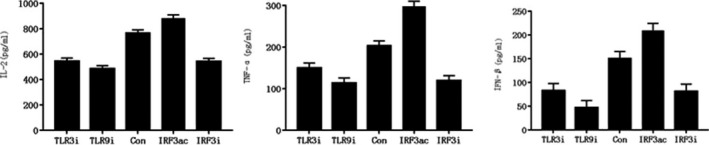
Expression of IL‐2, TNF‐alpha, and IFN‐beta in HaCaT cell model

The expressions of IL‐2, TNF‐alpha and IFN‐beta in TLR3 and TLR9 inhibitor groups were lower than those in the control group. IRF3 agonist was higher than that in the control group, RF3 inhibitor group was lower than that in the control group, and the difference was statistically significant (*P* < .05) (Figure 3).

### 3.4 WB analysis of TRAF3, IKK epsilon, and TBK1 expression in HaCaT cell model

**Figure 4:**
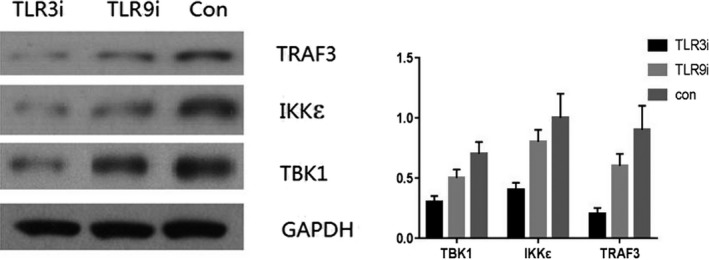
The expression of TRAF3, IKK epsilon, and TBK1 in HPV cells infected with HPV

The expression of TRAF3, IKK epsilon, and TBK1 in the control group was higher than that in the TLR3 and TLR9 inhibitor groups, and that in the TLR9 inhibitor group was higher than that in the TLR3 inhibitor group (*P* < .05), with significant difference (*P* < .05), as shown in Figure 4.

### 3.5 RT‐PCR analysis of IRF3 and IRF7 in HaCaT cell model

**Figure 5:**
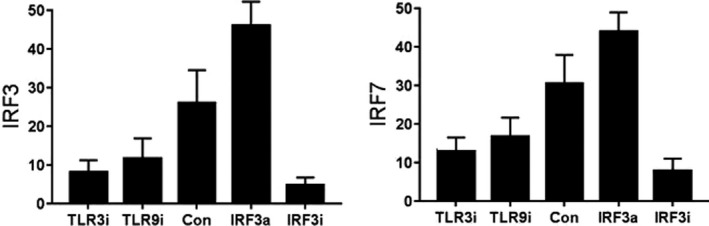
The expression of IRF3 and IRF7 in HaCaT cell model

The expression of IRF3 and IRF7 was lower in TLR3i and TLR9i groups than that in control group, higher in IRF3a group than that in control group, and much lower in IRF3i group than that in control group (*P* < .05). The difference was statistically significant (Figure 5).

## 4 DISCUSSION

Condyloma acuminatum has a high recurrence rate, which is related to the low cellular immune function of patients. Cellular immunity plays a major regulatory role in controlling the activation and regression of HPV and the response to treatment. T lymphocyte subsets play an important role in the body's anti‐virus cellular immunity, and the ratio of CD4^+^/CD8^+^ T lymphocyte is an important index to measure the cellular immune function.^8^, ^9^ T lymphocyte subsets interact with each other and maintain a certain balance in order to ensure the normal immune function. When the number and function of different lymphocyte subsets change abnormally, it will lead to the disorder of immune system and a series of pathological changes.^10^ CD4^+^ T cells can differentiate into Th1, Th2, Th17, and Treg subgroups in different cytokine environments. Th cells are the main participants in adaptive immune response. Studies have shown that the increase of CD4^+^ in peripheral blood of HPV patients suggests that the role of the immune system is enhanced. Th1 cells can secrete inflammatory cytokines such as IL‐2, IFN‐gamma, and TNF‐alpha to improve immunity.^11^, ^12^ Th2 secretes anti‐inflammatory factors, promotes the decline of immune cell function and apoptosis, causes the body's immune system to temporarily disable, and inhibits inflammatory response.^13^ Th17 and Treg, as two newly discovered lymphocyte subsets, play a central role in the body's autoimmune balance by inducing and maintaining immune tolerance. Th1/Th2 and Th17/Treg balance play an important role in the occurrence and development of inflammation and autoimmune diseases. In this study, the proportion of Th1, Th2, Th17, and Treg cells in the immune system of patients with HPV infection was the highest, indicating that the immune system responded to HPV infection by increasing the proportion of Th1 and Th17 to enhance the immune system's ability to clear the virus.^14^

TLR and its signal transduction pathway are important regulatory mechanisms for host cell recognition and response to viral infection recently discovered. Different cell signal transduction mechanisms are used to induce congenital immune response, which can directly affect the subsequent acquired immune system.^15^ TLRs are important model receptors for host cells to recognize viruses, which are mainly expressed in immune cells such as macrophages and dendritic cells. Studies have shown that the expression of TLR is enhanced after HPV infection. TLR pathway induces the production of type I interferon and pro‐inflammatory cytokines, and then induces the body's immune response. The expression of TLR3 and TLR9 is closely related to the clearance of HPV virus, which is highly expressed in cervical cancer patients.^16^, ^17^ In this study, the high expression of TLR3 and TLR9 in HaCaT cell model suggests that TLR3 and TLR9 play an important role in the immune response to HPV infection, and the mechanism may be through TLR signaling pathway. TLR is closely related to inflammatory factors, which can induce the body to respond effectively and rapidly to pathogens, promote cell activation, and initiate inflammatory signal transduction.^18^ In this study, under the action of TLR3 and TLR9 inhibitors, the corresponding inflammatory factors IL‐2, TNF‐alpha, and IFN‐beta showed low expression, and inflammatory factors IFN‐beta showed high expression under the action of IRF3 agonists, suggesting that LR3 and TLR9 were positively correlated with the levels of inflammatory factors and interferon in HPV‐infected cells, and TLR signaling pathway could regulate inflammation.

Interferon is a broad‐spectrum antiviral agent, which is the main antiviral defense and regulatory factor in the immune system. It can be divided into three types: IFN‐alpha (leukocyte), IFN‐beta‐(fibroblast), and IFN‐gamma (lymphocyte). The mechanism of virus clearance by immune system is very complex. There are many signaling pathways that can activate IFN. In the TLRs‐IKK e‐IRFs‐IFN signaling pathway, TLR combines with pathogen‐associated molecular patterns (PAMPs) to induce inflammatory response to eliminate pathogen infection, PAMP and IRF3 mediate to induce IFN expression^19^; IKKε/TBK1 is an IKK‐related activator, both of which participate in regulating IRF and play a role in IRF signaling pathway, phosphorylating IRF3 and IRF7, and activating IRF3 Function.^20^ In this study, after inhibiting TLR3 and TLR9, the expression of TRAF3, IKK epsilon, and TBK1 decreased, suggesting that TLR3 and TLR9 can affect IKK epsilon signaling pathway, and TLR3 and TLR9 can inhibit the expression of IKK epsilon. IRFs are a group of transcription factors that can regulate the expression of interferon (IFN) gene and play a role in the regulation of cellular immunity and proliferation. Finally, the high expression of IFN induces T‐cell differentiation, activates the body's specific adaptive immune pathway against pathogens, and completes virus clearance.^21^, ^22^ In this study, after inhibiting TLR3 and TLR9, the expression of IRF3 and IRF7 downstream decreased, suggesting that inhibiting TLR3 and TLR9 can inhibit the expression of IRF3 and IRF7 downstream, and TLR signaling pathway affects the expression of IRF signal downstream. The high expression of IRF directly promotes the high expression of IFN, thereby enhancing the immune clearance of HPV virus.

In summary, TLR3 and TLR9 are highly expressed in HPV‐infected HaCaT cells. Through TLRs‐IKK epsilon‐IRFs‐IFN signaling pathway, TLRs‐IKK epsilon‐IRFs‐IFN can induce high expression of inflammatory factors, IKK epsilon, IRFs, and IFN, and improve immune capacity.

## ETHICAL APPROVAL

The ethic approval was obtained from the Ethic Committee of Shanghai General Hospital, Shanghai Jiao Tong University School of Medicine.
